# Gz mediates the long-lasting desensitization of brain CB1 receptors and is essential for cross-tolerance with morphine

**DOI:** 10.1186/1744-8069-5-11

**Published:** 2009-03-10

**Authors:** Javier Garzón, Elena de la Torre-Madrid, María Rodríguez-Muñoz, Ana Vicente-Sánchez, Pilar Sánchez-Blázquez

**Affiliations:** 1 Instituto Cajal, CSIC, Dr. Arce 37, 28002 Madrid, Spain; 2 Centro de Investigación Biomédica en Red de Salud Mental, CIBERSAM, ISCIII, Dr. Arce 37, 28002 Madrid, Spain

## Abstract

**Background:**

Although the systemic administration of cannabinoids produces antinociception, their chronic use leads to analgesic tolerance as well as cross-tolerance to morphine. These effects are mediated by cannabinoids binding to peripheral, spinal and supraspinal CB1 and CB2 receptors, making it difficult to determine the relevance of each receptor type to these phenomena. However, in the brain, the CB1 receptors (CB1Rs) are expressed at high levels in neurons, whereas the expression of CB2Rs is marginal. Thus, CB1Rs mediate the effects of smoked cannabis and are also implicated in emotional behaviors. We have analyzed the production of supraspinal analgesia and the development of tolerance at CB1Rs by the direct injection of a series of cannabinoids into the brain. The influence of the activation of CB1Rs on supraspinal analgesia evoked by morphine was also evaluated.

**Results:**

Intracerebroventricular (icv) administration of cannabinoid receptor agonists, WIN55,212-2, ACEA or methanandamide, generated a dose-dependent analgesia. Notably, a single administration of these compounds brought about profound analgesic tolerance that lasted for more than 14 days. This decrease in the effect of cannabinoid receptor agonists was not mediated by depletion of CB1Rs or the loss of regulated G proteins, but, nevertheless, it was accompanied by reduced morphine analgesia. On the other hand, acute morphine administration produced tolerance that lasted only 3 days and did not affect the CB1R. We found that both neural mu-opioid receptors (MORs) and CB1Rs interact with the HINT1-RGSZ module, thereby regulating pertussis toxin-insensitive Gz proteins. In mice with reduced levels of these Gz proteins, the CB1R agonists produced no such desensitization or morphine cross-tolerance. On the other hand, experimental enhancement of Gz signaling enabled an acute icv administration of morphine to produce a long-lasting tolerance at MORs that persisted for more than 2 weeks, and it also impaired the analgesic effects of cannabinoids.

**Conclusion:**

In the brain, cannabinoids can produce analgesic tolerance that is not associated with the loss of surface CB1Rs or their uncoupling from regulated transduction. Neural specific Gz proteins are essential mediators of the analgesic effects of supraspinal CB1R agonists and morphine. These Gz proteins are also responsible for the long-term analgesic tolerance produced by single doses of these agonists, as well as for the cross-tolerance between CB1Rs and MORs.

## Background

The cannabinoid receptors belong to the G protein-coupled receptor (GPCR) superfamily and include at least two receptor types: CB1 and CB2 [1-3]. The systemic administration of endocannabinoids, such as anandamide, the synthetic agonist [(R)-(+)-[2,3-dihydro-5-methyl-3-(4-morpholinylmethyl) pyrrolo [1,2,3-de]-1,4-benzoxazin-6-yl]-1-naphthalenylmethanone] (WIN55,212-2) or the naturally occurring compound Δ^9^-tetrahydrocannabinol (THC), produces analgesia in rodent pain models. Unfortunately, long-term administration of agonists leads to a progressive decrease in the cannabinoid-mediated effects, a process referred to as analgesic tolerance [4,5], which persists for as long as 14 days [6]. The tolerance that follows repeated systemic administration of cannabinoids is caused by down-regulation and/or uncoupling of the receptors from the G proteins [1]. Most cannabinoids induce rapid internalization of their receptors via clathrin-coated pits [7], and long-term treatment leads to a significant down-regulation of CB1 receptors (CB1Rs). Indeed, an important fraction of the internalized receptors is transported to the lysosomal compartment for degradation, and the interaction between CB1Rs and G protein-associated sorting protein 1 (GASP1) plays an essential role in this process [8,9].

In the brain, CB1R is expressed at high levels in neural cells, whereas CB2R exists only at low levels [10]. Thus, CB1R appears to mediate the supraspinal effects of cannabinoid agonists. CB1R is found in the cerebral cortex, amygdala, hippocampus, basal ganglia, cerebellum, and brain areas involved in descending pain modulation, such as the periaqueductal gray matter (PAG), rostral ventromedial medulla (RVM), and the spinal cord [10]. This distribution is consistent with the effects of cannabinoids on emotional responses, cognition, memory, movement, and nociception [11-13]. At the molecular level, CB1R couples to pertussis toxin (PTX)-sensitive Gi/o proteins [14,15] and to certain pertussis toxin-insensitive G proteins, probably Gq/11 and Gz [16,17]. This receptor regulates the expression of immediate early genes and various cellular effectors, such as adenylyl cyclase, ion channels, mitogen-activated protein kinase, and focal adhesion kinase [1,3,18].

Interestingly, cannabinoids may be useful in controlling pathological pain that is resistant to conventional opioid therapies [19,20]. Although cannabinoids act independently of opioids to produce analgesia in rodents, similar brainstem circuitry seems to be involved [21]. Brain areas such as the caudate putamen, dorsal hippocampus, and substantia nigra are rich in both cannabinoid and opioid receptors, and the co-localization of both types of receptors has been described [10,22]. Loss of functional receptors leads to desensitization in both systems. Chronic treatment induces analgesic cross-tolerance between opioids and cannabinoids. This cross-tolerance, however, occurs without any change in the receptors of the other system [23,24]. This observation suggests that interactions can take place at the signal transduction/effector level. In nervous tissue, desensitization in response to single doses of opioids, such as morphine, occurs without the loss of surface receptors. However, in response to subsequent doses of morphine, the MOR becomes phosphorylated and undergoes internalization/recycling [25]. In contrast to what happens with the CB1R, few internalized MORs are destroyed in the lysosomal fraction [26,27]. Rather, most of the internalized MORs are re-inserted in the membrane, and tolerance to opioids that induce robust receptor internalization develops slowly [28,29].

A series of recent studies have increased our knowledge of the molecular mechanisms implicated in neural MOR signaling and desensitization. In nervous tissue, the C-terminus of MOR interacts with a signaling module consisting of histidine triad nucleotide binding protein 1 (HINT1) that is associated with the Gz regulatory proteins RGS17 (RGSZ2) and RGS20 (RGSZ1). The RGSZ proteins are specific GTPase-activating proteins (GAPs) of receptor-activated Gαz-GTP subunits. Thus, this HINT1-RGSZ signaling module is physically close to the MOR-regulated Gz proteins and helps to deactivate the agonist-activated Gαz-GTP subunits [30-32]. For opioids such as morphine, which induce little MOR internalization, neural-specific Gz proteins are essential to the desensitization of supraspinal antinociception.

Given the relevance of brain CB1Rs as analgesics, in the present study we sought to analyze supraspinal antinociception and its desensitization following icv administration of cannabinoid agonists. The results indicate that CB1Rs, like MORs [32], interact with the HINT1-RGSZ signaling module. Both receptors regulate Gz proteins to produce supraspinal analgesia, and this G protein is implicated in their desensitization and cross-desensitization.

## Results

### Single-dose analgesic desensitization of brain CB1 receptors: time required to recover the initial response

**Figure 1:**
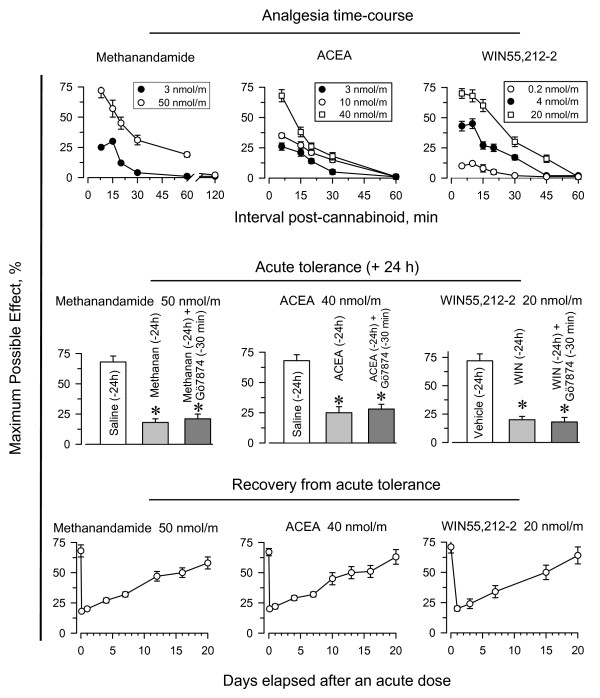
**Long-term supraspinal analgesic tolerance produced by agonists of brain CB1Rs**. *Upper panel*: Mice were injected icv with methanandamide, ACEA or WIN55,212-2 at the doses indicated, and analgesia was evaluated at various intervals post-injection using the "tail-flick" test with a thermal stimulus of a 52°C water bath. *Middle panel*: A priming dose of methanandamide (50 nmol), ACEA (40 nmol), WIN55,212-2 (20 nmol), or saline was administered icv to mice (n = 10 per group). After 24 h, single-dose tolerance was examined by administering a test dose of each agonist (same dose as the priming dose) to mice that had received either saline (open bars) or the priming doses (grey bars). In another set of assays, mice that had received the priming doses of the corresponding agonists were injected icv 24 h later with the PKC kinase inhibitor Gö7874 (1 nmol), and 30 min later with test doses of the cannabinoid agonists (dark grey bars) (n = 10 per group). The "tail-flick" test was conducted 10 min after agonist administration. Bars represent mean ± SEM. *Significantly different from the saline control group (*P *< 0.05). *Lower panel*: Recovery from the acute analgesic tolerance produced by the CB1R agonists. The "tail-flick" test was conducted 10 min after injection of the test dose. Data are expressed as mean ± SEM from groups of 8 mice.

The CB1R agonists methanandamide, ACEA, and WIN55,212-2 all produced dose-dependent antinociception in the tail-flick test when injected icv into mice (Fig. 1, upper panel); this antinociception was antagonized by the cannabinoid antagonist AM-251 (data not shown). These effects showed rapid onset, typically within 5 to 10 min post-injection, and then declined slowly. Doses of 50 nmol methanandamide, 40 nmol ACEA, or 20 nmol WIN55,212-2 produced comparable peak analgesic effects, which were approximately 70% of the maximum effect that can be observed in this test. Administration of an acute dose of these agonists caused a robust analgesic desensitization, and a second administration of either agonist 24 h after the priming dose produced almost no detectable analgesia (Fig. 1, middle panel). Because inhibiting PKC reverses single-dose analgesic tolerance to opioids such as morphine [see references in [33]], we tested the effect of injecting 1 nmol of the PKC inhibitor Gö7874 icv 30 min before the second dose of the agonists. Contrary to our expectations, this approach failed to rescue the analgesic effect of the cannabinoid agonists.

We next studied the interval required to recover the initial analgesic response. Mice received a desensitizing priming dose of the agonists, and were then divided into subgroups. Each subgroup received a second test dose of the agonist at different intervals after the priming dose. Notably, the CB1R desensitization caused by a single dose of the cannabinoid agonist lasted for several days and the recovery of the response took 2 or 3 weeks (Fig. 1, lower panel). This result differs from that observed in acute tolerance to morphine, which lasts only 3 or 4 days [34]. In this way, our results indicate that the CB1R agonists studied produce dose-dependent supraspinal analgesia, which leads to significant and long-lasting desensitization of the receptor. We then analyzed a series of mechanisms that could be responsible for this CB1R tolerance.

### Internalization of CB1Rs evoked in mouse brain by CB1R agonists

**Figure 2:**
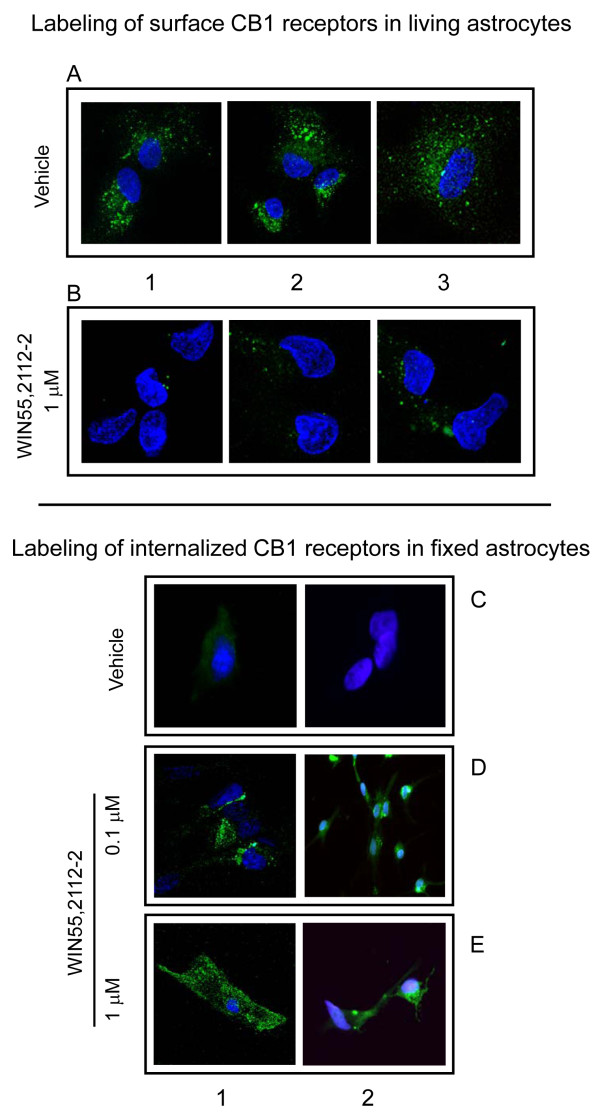
**WIN55,212-2 promotes internalization of CB1Rs in cultured astrocytes**. *Labeling of surface CB1Rs in living astrocytes*: astrocytes were incubated with WIN55,212-2 for 1 h, then CB1R antibodies (1:500) labeled with Alexa-488 were added to the cultures for 30 min. The antibodies were washed out and the tissue was fixed. The CB1R was labeled by antibodies targeting the first external loop (1L) or the N-terminus (Nt). Anti-CB1R Nt was used in A1, B1, A3, and B3; anti-CB1R 1EL was used in A2 and B2. Green = CB1R, blue = DAPI; magnification = 60×. *Labeling of internalized CB1Rs in fixed astrocytes*: astrocytes were first incubated with WIN55,212-2 for 1 h. Afterwards they were fixed and incubated with a solution containing 0.1% Triton X100. The CB1R antisera were added in this solution and incubated for 2 h. Anti-CB1R Nt was used in C1, D1, and E1; anti-CB1R 1EL was used in C2, D2, and E2. Details in Methods and Results.

A plausible explanation for the observed long-lasting single-dose desensitization of CB1Rs is that the agonists reduced the functional CB1Rs at the neural membrane. Because most of the neuronal CB1Rs are associated with synaptic terminals [35-37], our study was performed on the membrane fraction enriched in synaptosomes. Then, in order to directly evaluate the presence of the CB1Rs in the synaptosomal membrane, we developed two antibodies that recognize extracellular domains on the CB1 receptor: one is directed against a peptide sequence in the N terminus (Nt) and the other against the first extracellular loop (1EL). These antibodies were affinity-purified using the corresponding antigenic peptide sequence, and their ability to label the target proteins in neural cells was assessed. With this aim, living cultured astrocytes were incubated, in the absence of detergents, with the anti-CB1R antibodies, Nt or 1EL, which had been covalently attached to the fluorescent label, Alexa-488. Under conditions of no detergent, Triton X-100 does not disrupt the lipid bilayer and the IgGs do not penetrate into the living cells. In this case, both antibodies labeled only surface CB1Rs (Fig. 2A; Nt, A1 and A3; 1EL, A2). This labeling was not observed when the living astrocytes had been pre-treated with the cannabinoid agonist WIN55,212-2 (Fig. 2B). These observations indicate that both antibodies label extracellular epitopes of the CB1R and that WIN55,212-2 reduces its surface expression, probably by inducing receptor internalization.

**Figure 3:**
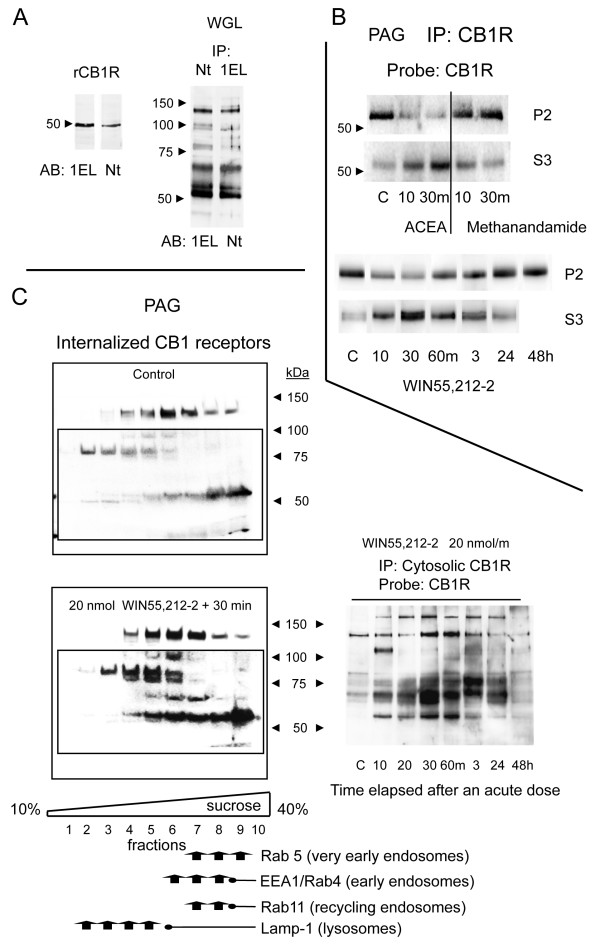
**Agonist-induced internalization of brain CB1Rs**. A. The recombinant CB1 receptor (rCB1R) was labeled by both Nt and 1EL antibodies. The CB1R in solubilized PAG synaptosomal membranes was precipitated with 1EL or Nt antibodies and then immunodetected using the other antibody, yielding a ladder pattern. This banding of CB1Rs was also observed in the purified glycosylated fraction of PAG membranes, indicating the presence of multiple glycosylated species. B. Cannabinoid agonists used at doses that desensitize analgesia caused the internalization of surface CB1Rs (P2) into the cytosol (S3). ACEA and WIN55,212-2 caused internalization, but methanandamide caused almost none. C. *Upper panel*: Presence of CB1Rs in the cytosolic fraction of brain tissue from mice that did not receive WIN55,212-2. The mice were killed and the PAG S3 fraction was subjected to subcellular fractionation in order to determine the presence of CB1Rs. *Lower panel*: Mice were injected icv with WIN55,212-2, then sacrificed 30 min later, and the PAG S3 cytosolic fraction was analyzed for the presence and distribution of internalized CB1Rs. Subcellular markers: Rab5 (BD 610281), EEA1 (early endosome antigen I; BD 610456), Rab4 (BD 610888), Rab11 (BD 610656), Lamp-1 (lysosomal-associated membrane protein I; BD 61043). *Right panel*: The PAG cytosolic fraction was analyzed at various time intervals following administration of WIN55,212-2 in order to test for the presence of the CB1R.

In order to detect the internalized CB1Rs, the living astrocytes were exposed to WIN55,212-2 and then incubated with a fixative solution that modifies extracellular sequences of surface proteins, such as the CB1Rs. However, if the integrity of the cell membrane is preserved, the fixative does not reach and alter the sequences of the cytosolic proteins. The anti-CB1R antibodies were then added in a medium containing 0.1% Triton X-100 to disorganize the lipid membrane. In doing so, the antibodies failed to bind the corresponding epitopes on the surface CB1Rs (compare Fig. 2A and 2C), but reached the intact epitopes on the internalized CB1Rs (Nt, D1 and E1; 1EL, D2 and E2). Therefore, WIN55,212-2 at concentrations of 0.1 μM and 1 μM, promoted the internalization of the CB1Rs (Fig. 2C and 2D). These results indicate that the Nt and 1EL antibodies bind to CB1Rs. Moreover, both antibodies recognized the recombinant CB1R protein in immunoblotting assays (Fig. 3A).

The supraspinal analgesic effects of icv-injected opioids and cannabinoids occur mainly through their respective binding to MORs and CB1Rs in the midbrain [10,12,38-40]. These substances act on the PAG to RVM connection, where certain neurons carrying these receptors in the RVM project down to the substantia gelatinosa in the dorsal horn of the spinal cord and reduce the intensity of the ascending nociceptive signals [41,42]. Therefore, to compare the analgesic effects with molecular events, we examined the discrete neural structure, the PAG. The neural CB1R has been described as heavily glycosylated [43]. Consequently, our anti-CB1R antibodies revealed a stepladder pattern, reflecting differently glycosylated populations. The covalently bound sugar branches prevent CB1R from adopting the globular shape that is expected for proteins subjected to sodium dodecyl sulfate polyacrylamide gel electrophoresis (SDS-PAGE). Thus, sugars reduce the mobility of this protein through the pores of the gel and produce the observed bands (ladder steps), depending on the kind of sugar or the extent of the glycosylation [44]. This stepladder pattern was also observed when the receptor was immunoprecipitated from the synaptosomal glycosylated protein fraction (obtained by WGL affinity chromatography) and analyzed using the other CB1R antibody (Fig. 3A). In neurons, glycosylation of GPCRs is very common and produces similar patterns after SDS-PAGE (e.g., μ- and δ-opioid receptors) [[45] and references therein].

For the sake of simplicity, we studied the agonist-induced internalization of brain CB1Rs by focusing on the 55- to 60-kDa band. In these assays, the cannabinoid agonists were used at acute doses that produced a long-lasting analgesic tolerance. Early after icv ACEA or WIN55,212-2 administration and during their analgesic time-courses, the level of cytosolic CB1Rs increased and the level of surface CB1Rs decreased. This trend gradually changed after an additional 48 h, such that the presence of this receptor in the cytosol and at the cell surface had returned to the levels observed in the absence of agonist (Fig. 3B and 3C). In contrast, methanandamide induced a low to moderate internalization of the CB1R (Fig. 3B). However, over several days, the mice remained refractory to the analgesic effects of additional doses of all these agonists.

A significant fraction of the internalized CB1Rs is shuttled into lysosomes, where the receptors are degraded. Therefore, the recovery of the surface CB1Rs observed after a single dose of WIN55,212-2 indicates that CB1R turnover takes about 28 h. In the absence of an exogenous agonist, some of the cytosolic CB1R staining is not associated with lysosomes and may indicate synthesis (Fig. 3C). In contrast, MOR, which has a turnover rate of 10 to 14 days, is barely detectable in the cytosol in the absence of agonist treatment [25]. After WIN55,212-2 challenge, CB1Rs were internalized and their levels in early endosomes and lysosomes increased. These findings indicate that the CB1R is being lysosomally degraded [9]. Based on the antibody staining pattern, it appears that the transition between early endosomes and lysosomes depends on the level of CB1R glycosylation. The 55- to 60-kDa band remained in the fraction containing early and recycling endosomes longer than did the bands representing more slowly migrating proteins, which rapidly associated with lysosomes (Fig. 3C). This also seems to be the case for the opioid receptors: the heavily glycosylated forms had the fastest turnover [46].

Methanandamide induced minimal internalization of CB1Rs in the brain, but it did promote analgesic desensitization. Moreover, surface synaptosomal CB1Rs depleted by WIN55,212-2 and ACEA recovered long before the analgesic response was restored. These observations indicate that the acute analgesic tolerance produced by cannabinoid agonists given by the icv route is not due to the loss of surface receptors.

### Agonists of CB1Rs do not promote stable transfer of G proteins to RGS proteins

**Figure 4:**
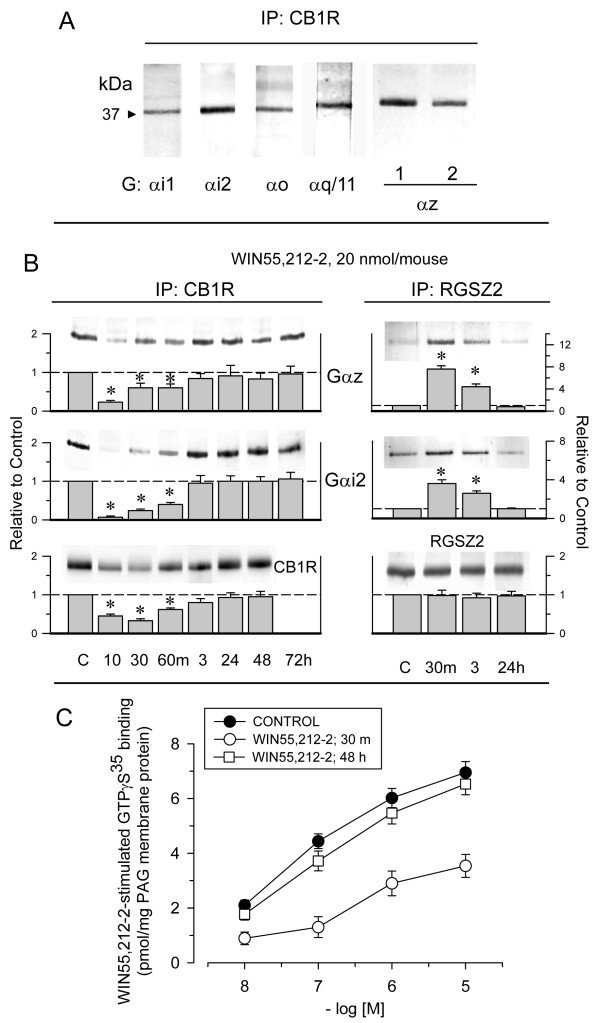
**WIN55,212-2 promotes reversible transfer of CB1R-regulated G proteins toward RGSZ2 proteins**. A. Gα subunits co-precipitated with CB1Rs in PAG membranes. These proteins were assayed using antibodies to different Gα subunits. Gαz was studied using an antibody targeting the helical domain (1) and an antibody targeting the C-terminus (2). B. Groups of mice received a single icv injection of WIN55,212-2 (20 nmol) and were killed at the time points indicated. For each time point studied the PAG structures from 6 to 8 mice were pooled. The CB1R, RGSZ2, and associated proteins were then studied in PAG membranes. The immunosignals associated with these signals (average optical density of the pixels within the object area/mm2; Quantity One Software, BioRad) were expressed relative to the levels observed for the control group (attributed an arbitrary value of 1). Each bar is the mean ± SEM of three assays performed on PAG samples obtained from independent groups of mice. *Significantly different from the PAG control group (*P *< 0.05). C. Stimulated [^35^S]GTPγS binding by WIN55,212-2 in PAG membranes from mice injected with a cannabinoid agonist. Membranes from mice treated with vehicle (control; closed symbols) or exposed to WIN55,212-2 (20 nmol/mouse; open symbols) were obtained 30 min and 48 h after icv injection and incubated in [^35^S]GTP*γ*S (0.1 nM) and increasing concentrations of WIN55,212-2. *Significantly different from the PAG control group (*P *< 0.05).

Receptor desensitization can be achieved without affecting the levels of surface receptors. This has been observed for morphine, which promotes high analgesic tolerance with no loss of surface MORs [25]. In the brain, most cases of tolerance to morphine occur as a result of depletion of the MOR-regulated G proteins, which are transferred to RGS proteins belonging to the R7 and Rz subfamilies [25,47,48]. In the mouse brain, the CB1R is associated with different classes of G proteins. In the PAG, this receptor co-precipitates with PTX-sensitive Gi and Go as well as with PTX-insensitive Gq/11 and Gz (Fig. 4A). WIN55,212-2 (20 nmol), which induces the internalization of PAG CB1Rs, also promoted some transfer of Gαi and Gαz subunits from CB1Rs toward RGSZ2 proteins. However, this association was only transitory, and when the CB1Rs gradually returned to the membrane, they reassociated with the G proteins (Fig. 4B, C). This is not the case for morphine, however, which promotes a long-term transfer of Gi/z proteins toward RGSZ2 proteins and thus a persistent decrease in the signaling capacity of MORs. Interestingly, the opioid agonist [D-Ala^2^, *N*-Me-Phe^4^, Gly-ol^5^]-encephalin (DAMGO), which stimulates robust internalization of MORs, also promotes temporary association of G proteins with RGSZ2 [25]. However, DAMGO produces low levels of analgesic tolerance, whereas WIN55,212-12, ACEA, and methanandamide induce a significant and long-lasting tolerance. These observations indicate that cannabinoid tolerance is not mediated by reductions in CB1R-regulated transduction.

### Influence of G proteins and RGS proteins in the desensitizing capacity of cannabinoids

We next explored whether cannabinoid desensitization is caused by post-receptor events triggered by certain G proteins and/or RGS proteins. To accomplish this, we used antisense oligodeoxynucleotides (ODNs) targeting mRNAs encoding specific Gα subunits and RGS proteins in order to reduce the expression of these proteins. The ODNs used have all been extensively characterized as effective and selective in reducing expression of the murine target protein and are referred to in the corresponding studies: Gαz subunit [49,50]; Gαi2 subunit [49,50]; Gαq/Gα11 [51]; RGS9-2 [48,52]; RGSZ2 [47]; PKCI/HINT1 [32].

**Figure 5:**
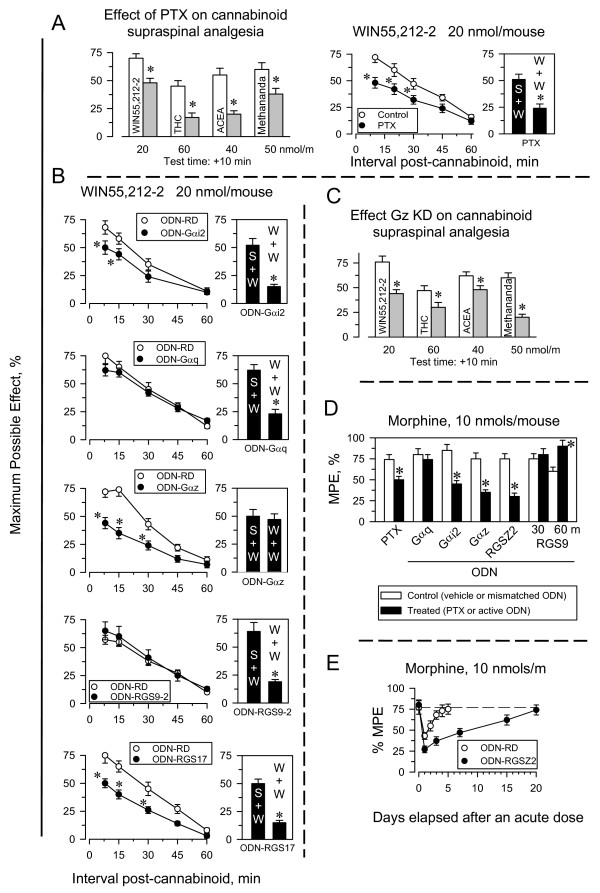
**Neural-specific Gz proteins mediate supraspinal analgesic desensitization produced by CB1R and MOR agonists**. A. Effect of Pertussis toxin (PTX) on the supraspinal analgesia produced by cannabinoid agonists. PTX injected icv to mice (0.5 μg) was given 6 days before the agonist [55]. Left panel: Open bars indicate controls; gray bars indicate mice treated with PTX. Right panel: The influence of PTX on the analgesic time-course of 20 nmol WIN55-212-2 and on its single-dose tolerance was studied. Either saline (S) or the priming dose of the agonist (W) was administered icv to mice. After 24 h, all groups received an icv test injection of WIN55,212-2 (S+W; W+W), and the analgesic effects were evaluated 10 min later. Values are mean ± SEM for groups of 8–10 mice. *Significantly different from the control group injected with saline instead of the priming dose of WIN55,212-2 and also receiving the test dose of agonist (S+W) (*P *< 0.05). B. Time-course for the supraspinal analgesic effects of WIN55,212-2 was evaluated in mice that had been subjected to different icv treatments. Reduction in the levels of certain proteins was attained using oligodeoxynucleotides directed against Gαq [51], Gαi2 [49,50], Gαz [49,50], RGS9 [48,52], and RGSZ2 [47]. ODN-RD stands for the control mismatched ODN. Values are mean ± SEM from groups of eight mice. *Significantly different from the control group injected with either saline or the corresponding control ODN (*P *< 0.05). The effect of these treatments on the single-dose tolerance produced by 20 nmol WIN55,212-2 was studied as described above. C. The analgesic effects of WIN55,212-2, THC, ACEA and methanandamide was studied in mice with impaired expression of Gαz subunits. D. A parallel study was carried out with morphine in mice subjected to the indicated treatments. Data corresponding to the peak effect of morphine at 30 min are shown. Findings from RGS9 knockdown mice are also shown at 60 min. Values are mean ± SEM from groups of eight mice. *Significantly different from the control group injected with saline (for PTX) or the corresponding control ODN (*P *< 0.05). E. Recovery from the acute analgesic tolerance produced by morphine in control mice and mice with reduced expression of RGSZ2 protein. For details see Fig. 1, lower panel.

The supraspinal analgesia evoked by CB1R agonists was only partially sensitive to the effect of PTX [53]. This result suggests the participation of both PTX-sensitive and PTX-insensitive G proteins in supraspinal analgesia mediated by CB1Rs. In our study, the analgesia produced by WIN55,212-2 and methanandamide was more resistant to the blocking effect of PTX than that of ACEA or THC (Fig. 5A). Consistent with this observation, knockdown of the PTX-sensitive Gαi2 proteins produced little change in the activity of WIN55,212-2. Of the PTX-resistant G proteins, knockdown of Gαz, but not Gαq, significantly reduced the analgesic effects of WIN55,212-2 (Fig. 5B). Besides, the supraspinal antinociception evoked by methanandamide decreased when expression of Gαz was reduced. This reduction was only moderated for THC and ACEA (Fig. 5C). For all of the agonists studied, the desensitization that followed was not significantly altered by treating the mice with PTX (shown for WIN55,212-2, Fig. 5A). Notably, these Gαz knockdown mice did not develop acute tolerance to the analgesic effects of cannabinoids when evaluated 24 h (Fig. 5B middle panel), 48 h and 72 h later (not shown). The antinociceptive activity of morphine is partially sensitive to PTX, and Gαi2 subunits play a role (Fig. 5D) [50,54,55]. The morphine analgesic effect is independent of Gαq subunits but strongly decreases after Gαz knockdown [50,54,56].

The RGS9 protein is a negative regulator of morphine activity and, in its absence, morphine has an increased analgesic effect and induces less tolerance [52,57]. Nevertheless, the absence of RGS9 did not affect the activity of WIN55,212-2 or its capacity to produce CB1R desensitization. On the other hand, reduced RGSZ2 expression caused increased Gz signaling and rapid desensitization of MORs [47], which lasted for more than 2 weeks (Fig. 5E). This pattern is consistent with CB1R desensitization produced by the acute doses of icv cannabinoid agonists (see Fig. 1, lower panel). Therefore, the CB1R agonists appear to produce long-lasting acute analgesic desensitization by activating a signaling pathway in which Gz proteins play an essential role.

### Cross-tolerance between CB1Rs and MORs

**Figure 6:**
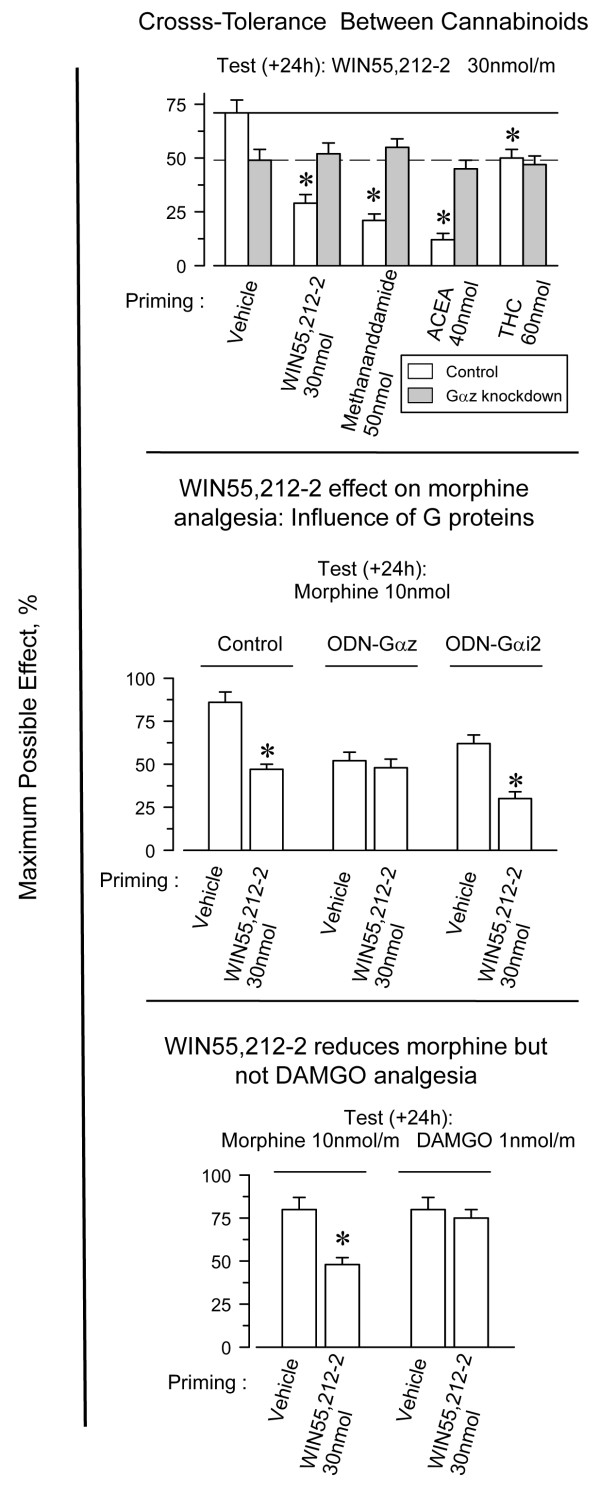
**Role of Gz proteins in the ability of WIN55,212-2 to reduce morphine analgesia**. *Upper panel*: Cross-tolerance between CB1R agonists in wild type and Gαz knockdown mice. A priming dose of WIN55,212-2 (icv; 30 nmol) was administered to the mice, and their response to WIN55,212-2, methanandamide, ACEA or THC was evaluated 24 h later. Values are mean ± SEM from groups of eight mice. *Significantly different from the control group injected with the vehicle instead of the priming dose of WIN55,212-2 and also receiving the test dose of this agonist *(P < 0.05). Middle panel*: WIN55,212-2 does not reduce morphine analgesia in mice showing reduced expression of Gαz subunits. The activity of WIN55,212-2 on morphine-evoked antinociception was studied in mice showing reduced expression of either Gαi2 or Gαz subunits. *Significantly different from the control group injected with the vehicle instead of the priming dose of WIN55,212-2 *(P < 0.05). Lower pane*l: Effect of WIN55,212-2 on MOR-mediated analgesia. A priming dose of WIN55,212-2 (icv; 30 nmol) was administered to mice 24 h before testing the supraspinal analgesic response to morphine and DAMGO. Control mice received vehicle instead of the cannabinoid agonist. *Morphine displays significantly lower analgesic effect on mice that previously received WIN55,212-2 instead of vehicle (*P *< 0.05).

WIN55,212-2 desensitized its own analgesic response and, as expected for agonists sharing the receptor, it was also desensitized by other CB1R agonists, such as methanandamide, ACEA, and THC. This cross-desensitization produced by acute doses of cannabinoid agonists was not observed after reducing the expression of Gαz subunits (Fig. 6, upper panel). We explored whether the interaction between CB1R agonists and morphine was mediated by the activation of Gz proteins. WIN55,212-2 diminished the analgesic response of morphine, and this reduction was observed even after 5 days of icv cannabinoid injection. Notably, in Gαz knockdown mice, WIN55,212-2 produced no cross-tolerance to morphine analgesia. However, the connection between CB1R and MOR persisted when the expression of Gαi2 was reduced (Fig. 6, middle panel). Thus, Gz plays a key role in impairing the activity of the CB1R agonist, WIN55,212-2, on morphine-promoted analgesia. An interesting result is that WIN55,212-2 failed to reduce the response to DAMGO (Fig. 6, lower panel).

**Figure 7:**
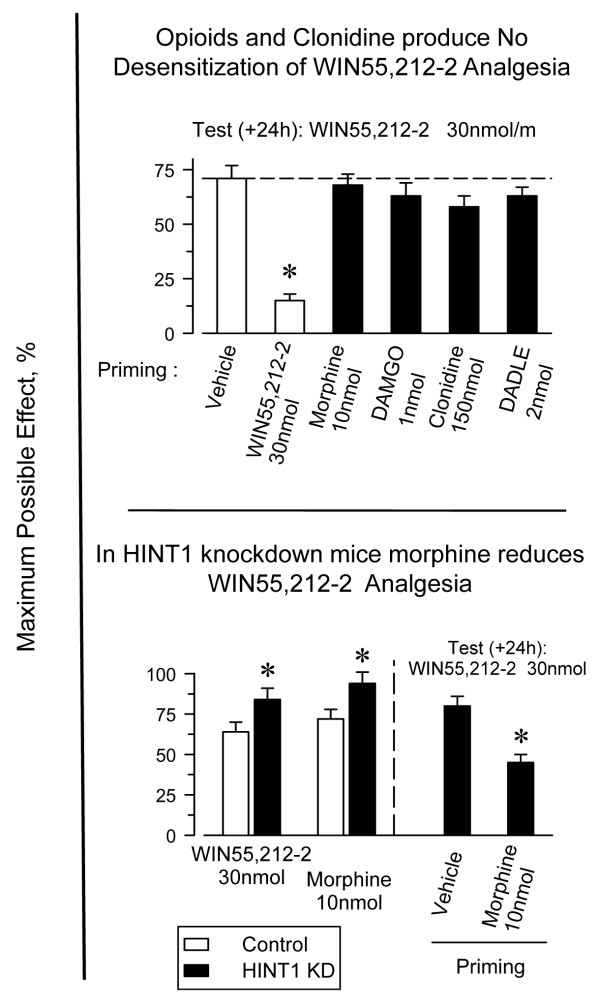
**Morphine reduces WIN55,212-12 analgesia in mice that do not express HINT1 proteins**. *Upper panel*: Groups of eight mice each were injected icv with the opioids morphine, DAMGO, DADLE, and clonidine at the indicated doses, and the analgesic effect of WIN55,212-2 was assessed 24 h later. *Significantly different from the control group injected with the vehicle instead of the priming dose of WIN55,212-2 *(P < 0.05). Lower panel*: The absence of HINT1 enables morphine to reduce the analgesic effects of WIN55,212-2. *Significantly different from the control animals (open bar) or from the group injected with the vehicle instead of the priming dose of morphine (*P *< 0.05).

Acute administration of opioids at their analgesic ED_80_, regardless of whether their ability to activate Gz proteins was strong (morphine), moderate (DAMGO), or low (DADLE) [51,58], produced no reductions in the analgesic effects of WIN55,212-2. Clonidine, an agonist of α2 adrenoreceptors that activates Gz proteins [59], also failed to alter the activity of the CB1R agonist (Fig. 7, upper panel). These results indicate that acute desensitization operates only in the direction from the CB1R toward the MOR. It may be that MORs as well as α2A receptors efficiently regulate Gz-mediated signaling, thereby promoting little desensitization of Gz target effectors. In fact, MOR tolerance produced by an acute dose of morphine lasted for only 3 or 4 days (e.g., Fig. 5D), whereas that of CB1R agonists persisted for more than 14 days. Consistent with this idea, deregulation of receptor-activated Gαz-GTP subunits through reduction in the expression of its GAP, the RGSZ2 protein, leads to profound desensitization of morphine analgesia [47,54]. Under these circumstances, the tolerance evoked by a single dose of morphine lasted as long as that observed following acute icv doses of the CB1R agonists (see Fig. 5E).

To add consistency to these results, we considered the possibility that morphine diminishes CB1R activity after deregulation of Gz proteins. However, after knockdown of RGSZ2 proteins, the analgesic effects of both morphine and WIN55,212-2 were greatly reduced; thus, no interaction could be reliably studied. To circumvent this difficulty, we considered an alternative approach to deregulate the receptor-activated Gz proteins. The HINT1-RGSZ signaling module on the C-terminus of MORs helps control the MOR-induced activation of Gz proteins [30-32]. The absence of HINT1 dissociates RGSZ proteins from MORs and reduces the deactivation of the morphine-activated Gαz-GTP subunits. Consistent with this, morphine displays a higher analgesic potency in HINT1 knockout/knockdown mice [31,32]; we also observed an increased analgesic effect of WIN55,212-2 in these mice (Fig. 7, lower panel). These increases in morphine and WIN55,212-2 analgesic effects were followed by a greater tolerance to their respective analgesic effects. However, this analgesic tolerance was of a lesser magnitude than that observed when RGSZ2 was depleted [31,32] and permitted the study of the influence of morphine on WIN55,212-2 antinociceptive effects. Most relevant, in those HINT1 knockdown mice, morphine effectively reduced the analgesia evoked by WIN55,212-2 (Fig. 7, lower panel).

### The neural CB1R interacts with the HINT1-RGSZ module

**Figure 8:**
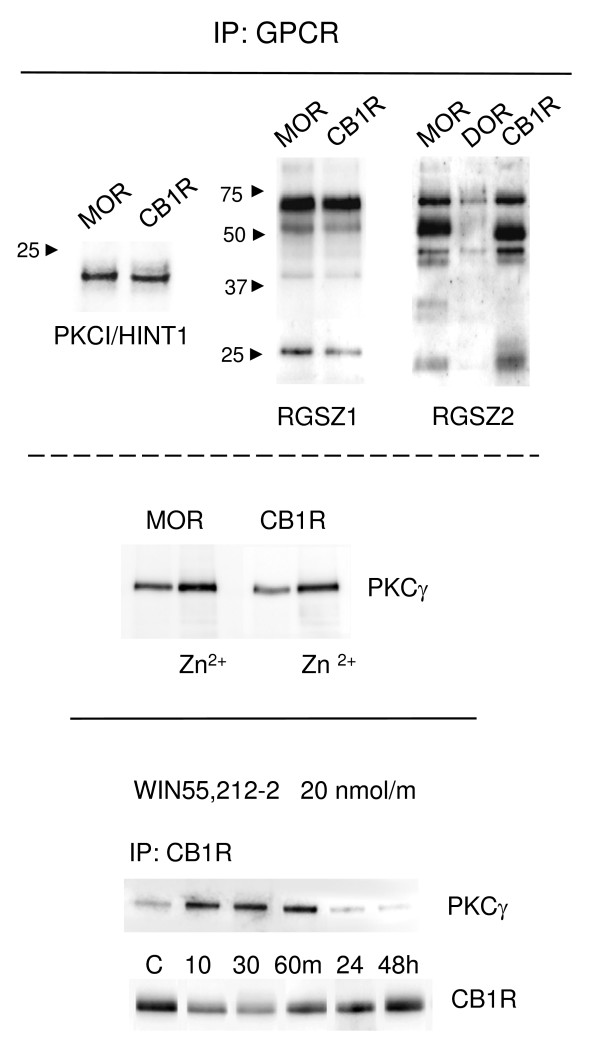
**The CB1R carries the HINT1-RGSZ signaling module**. *Upper panel*: In solubilized PAG synaptosomal membranes, MOR and CB1R co-precipitated PKCI/HINT1 and RGSZ proteins. In contrast, the δ-opioid receptor (DOR) showed little association with these proteins. *Middle panel*: Zinc increases the association of PKCγ with PAG MOR and CB1R. PAG synaptosomes were solubilized and incubated with zinc (30 nM) for 4 h at 4°C. MORs and CB1Rs were immunoprecipitated, and co-precipitation of PKCγ was evaluated. *Lower panel*: WIN55,212-2 promotes the recruitment of PKCγ to the CB1 receptor. Mice were injected icv with a desensitizing dose of 20 nmol WIN55,212-2. The CB1 receptors were immunoprecipitated (IP) from PAG synaptosomes (P2) obtained at the indicated intervals following agonist administration. For each interval, the PAG from six to eight mice were pooled. Since this dose of WIN55,212-2 promotes internalization of PAG CB1Rs, equal loading was verified by examining the signals obtained by immunodetection of the heavy chain of the anti-CB1R IgGs. IgGs were detached from the immunoprecipitated CB1 receptors, processed in parallel gel/blots, and detected using the appropriate secondary antibody. Co-precipitation studies were performed under non-denaturing conditions, and the MOR- and CB1R-associated proteins were immunodetected with antibodies directed against PKCI/HINT1, PKCγ, RGSZ1, and RGSZ2.

The results of this study so far indicate that brain CB1R regulates neural-specific Gz proteins. We next analyzed the possible linkage of the CB1R to the HINT1-RGSZ module originally described for the MOR [30], which also couples to Gz proteins [49]. We found that HINT1 co-precipitates with both MORs and CB1Rs. Moreover, both receptors co-precipitated RGSZ1 and RGSZ2 proteins. However, the δ-opioid receptor, which does not regulate Gz proteins in brain tissue [49], precipitated almost no RGSZ proteins (Fig. 8, upper panel). The pattern of RGSZ bands detected resembled a stepladder due to the heterogeneity in glycosylation and sumoylation [60].

As described for the HINT1-RGSZ module when bound to the MOR C-terminus [32], the CB1R also increased its association with the ser/thr kinase, PKCγ, in the presence of free zinc ions (Fig. 8, middle panel). Thus, administration of morphine promotes increases in the association of PKCγ with the MOR-HINT1-RGSZ complex, probably in order to phosphorylate and uncouple the receptor from downstream G proteins. Likewise, WIN55,212-2 injected icv into the mice also promoted an increase in the association of CB1R-HINT1-RGSZ with this kinase. This increased association of PKC was most often observed when the association of G proteins with CB1R decreased (compare Fig. 4B and lower panel of Fig. 8). Thus, the recruited PKCγ plays a role in uncoupling the CB1R from regulated transduction before it becomes internalized.

## Discussion

This study has shown that acute icv administration of cannabinoids promotes a long-lasting and robust desensitization of supraspinal CB1Rs, which could be mediated by post-receptor events. The analgesic tolerance that is observed after ip or sc administration of these substances usually requires repeated injections and affects both CB1 and CB2 receptors at spinal and supraspinal levels [10,61]. This long-term cannabinoid administration produces CB1R desensitization and down-regulation [6]. Specifically, supraspinal CB1R expression diminishes, as does the ability of systemic cannabinoids to induce hypoactivity, hypothermia, and antinociception [5,8,9]. Up to 2 weeks are required to recover the initial levels of CB1Rs in the synaptosomal membrane as well as the analgesic response to cannabinoids [5,6,9,62]. Therefore, the analgesic tolerance that follows the repeated systemic administration of cannabinoids can be explained in terms of the loss of surface CB receptors.

Brain CB1Rs, however, desensitize in response to acute doses of agonists; this cannot be explained merely in terms of a permanent loss of receptors. The effect of a single icv-injection of ACEA or WIN55,212-2 on surface CB1Rs is certainly brief. During the analgesic time-course of these agonists, the CB1Rs decreased in the PAG membrane by 60–70%. Most of the internalized CB1Rs bind to GASP1 and are then degraded in the lysosomal compartment [8,9]. After the analgesic effects of single doses of the cannabinoids cease, the CB1Rs are gradually restored to the surface, probably by both the recycling of a portion of the internalized receptors and the insertion of newly synthesized receptors. As a result, 24 h or 48 h later, the presence of CB1Rs in the membrane is comparable to that seen before the agonist challenge. During this time, the restored CB1Rs become coupled to G proteins, but the analgesic response takes a significantly longer time to be restored: about 14 days. Most relevant, this tolerance is also promoted by cannabinoids such as methanandamide, which cause almost no loss of surface CB1Rs. Thus, it is likely that the analgesic desensitization promoted after several days of systemic treatment with cannabinoids primarily affects receptors at the spinal and peripheral levels, and the associated downregulation of the supraspinal CB1Rs, about 30–50% [5,62], may be secondary to these effects.

There is compelling evidence that the CB1R couples to and regulates both PTX-sensitive Gi/o proteins and PTX-insensitive Gq/11 and Gz proteins. Thus, the endocannabinoid, 2-arachidonoylglycerol, protects neurons by limiting cyclooxygenase-2 expression, an effect mediated by PTX-sensitive G proteins [15]. WIN55,212-2 shows a more complex pattern of receptor activation. Whereas this agonist affects acetylcholine release in the hippocampus through a PTX-sensitive mechanism [63], in cultured hippocampal neurons it promotes increases in intracellular calcium via CB1Rs and the PTX-insensitive Gq protein. Interestingly, the latter effect is not reproduced by other cannabinoids, such as THC, CP55,940, 2-arachidonoylglycerol, or methanandamide [16]. These results indicate that after binding the CB1R, cannabinoids may determine the class(es) of G proteins to be activated. Indeed, in a cell line derived from human trabecular meshwork, which is an ocular tissue, WIN55,212-2 was shown to increase intracellular calcium via CB1R and Gq/11 proteins and to increase ERK1/2 phosphorylation via PTX-sensitive Gi/o proteins. In this system, CP55,940 and methanandamide produced the same effects, but they acted via PTX-sensitive Gi/o proteins [17]. Therefore, the CB1R, like the MOR, couples to a series of PTX-sensitive and -insensitive G proteins, and the agonists determine the pattern of G protein activation [56,58,64].

Brain CB1Rs mediate the production of analgesia via PTX-sensitive and PTX-insensitive G proteins [[53] and present study]. The spinal-mediated analgesic action of cannabinoids is mostly mediated via Gi/o proteins. Intrathecal administration of PTX also abolishes the analgesia evoked by icv cannabinoids, indicating that the descending pathways triggered by these substances act at the spinal level through receptors coupled to Gi/o proteins [65]. Signaling via the neural-specific PTX-insensitive Gz protein appears to occur more at the supraspinal level [55,66]. In fact, supraspinal analgesia mediated by MORs has an important Gz component [56]; at the spinal level, in contrast, PTX abolishes most MOR-mediated analgesia [66,67]. Consistent with this observation, the levels of expression of specific regulators of activated Gαz subunits, GAPs, RGSZ1, and RGSZ2, are lower in the spinal cord than in the midbrain [47,54].

Activation of Gz proteins mediates long-lasting analgesic desensitization of supraspinal CB1Rs. Cannabinoid agonists, such as methanandamide, which apparently do not activate Gq/11 proteins [16,17], produced desensitization of CB1Rs via activation of Gz proteins. Therefore, it seems that agonists that trigger activation of Gi/o proteins via CB1Rs also activate the PTX-insensitive Gz protein. The unique biochemical and regulatory properties of Gαz subunits account for their strong ability to desensitize GPCR signaling events. The Gz transducer protein, like Gi/o proteins, regulates adenylyl cyclase activity and the gating of certain K^+ ^channels. Gαz, however, is predominantly confined to neuronal cells. The rate of Gαz-GTP hydrolysis is as much as 200-fold slower than that of Gαs-GTP and Gαi-GTP. Therefore, Gz may be resistant to inactivation after receptor activation unless external factors accelerate the rate of Gαz-GTP hydrolysis, much the same way that the GAPs do for many Ras-like proteins. Therefore, inadequate control of Gαz signaling may easily lead to over-regulation of target effectors and subsequent desensitization [47]. Thus, deactivation requires the assistance of specific GAPs to augment the rate of hydrolysis and thus release effector(s) from continuous regulation.

The RGS-Rz subfamily bears the primary responsibility for regulating Gz, and the C-terminus of the MOR associates with a signaling module consisting of HINT1-RGSZ, which helps deactivate MOR-activated Gαz-GTP subunits [30]. This study has shown that brain CB1Rs regulate Gz proteins and associate with the HINT1-RGSZ signaling module, which is involved in the zinc-mediated recruitment of PKCγ [32]. Indeed, PKC has been implicated in the desensitization of CB1Rs by phosphorylation of a serine residue (S317) in the third internal loop [68]. We have observed the in vivo recruitment of PKCγ toward the HINT1-RGSZ module at the C-terminus of CB1R during the intervals when the receptor is uncoupled from regulated transduction. However, the inhibition of this kinase did not prevent the development of acute tolerance, suggesting that other post-receptor mechanisms operate in this process. Most relevant, deregulation of this module brings about increased Gz signaling at the corresponding effector(s) and the development of profound analgesic desensitization of brain MORs [47,50,54] and CB1Rs (present study). In contrast, depletion of Gz proteins reduces the analgesic desensitization produced by icv injection of various doses of morphine [50] and also abolishes acute desensitization of brain CB1Rs and cross-tolerance with morphine. A single icv injection of morphine produces desensitization that lasts for approximately 3 days; however, the cannabinoid agonists studied here desensitized CB1Rs for more than 14 days. Because both of these effects were mediated by the activation of Gz proteins, this observation indicates that CB1R-activated Gz proteins are controlled less efficiently than those activated by the MOR. In agreement with this idea, disruption of the HINT1-RGSZ module led acutely administered morphine to promote a profound and long-lasting desensitization of brain MORs, and most relevant, impaired the analgesic activity of CB1R agonists. Therefore, disruption of Gz regulation brings about a bidirectional supraspinal cross-tolerance between acute doses of morphine and cannabinoids, similar to that attained through chronic and systemic administration of the respective MOR or CB1R agonists [23,24].

Morphine poorly internalizes MORs and promotes strong analgesic desensitization by stimulating permanent transfer of a part of the receptor-regulated G proteins toward RGS proteins belonging to the R7 and Rz subfamilies [25,47,48,50]. In contrast, DAMGO produces a robust internalization and recycling of MORs, a transient transfer of G proteins toward the RGS proteins, and a low level of analgesic tolerance [25]. Because icv-injected cannabinoids facilitated a reversible transfer of Gα subunits toward RGSZ2 proteins and because the membrane levels of CB1Rs were almost restored within 24 h of their initial challenge, one should expect the resensitization of the analgesic response to these substances, as with DAMGO. However, our results reveal a long-term supraspinal analgesic tolerance even after the CB1R reassociates with these G proteins. This apparent divergence between DAMGO and cannabinoids in the production of tolerance can be explained in terms of the classes of G proteins activated by these agonists after binding to their respective supraspinal receptors. Thus, the analgesic effects of WIN55,212-2 are mediated mostly by Gz proteins, whereas those of DAMGO require Gi/o proteins and, to a lesser extent, Gz [51,56,58,64]. In the absence of Gz activation, the cannabinoids behave as DAMGO, promoting low levels of analgesic tolerance. Therefore, the desensitizing capacity of Gz proteins on post-receptor events predominates over the resensitization caused by reinsertion and G protein-coupling of the internalized CB1Rs in the neural membrane. Thus, it is the coincidence of WIN55,212-2 and morphine at Gz proteins that accounts for their cross-desensitization, whereas the poor regulation of this Gz by DAMGO explains why WIN55,212-2 fails to impair DAMGO-evoked analgesia.

Thus, an inefficient Gαz-GTP deactivation results in desensitization of brain MORs and CB1Rs, suggesting a post-receptor mechanism that appears to be regulated by Gz proteins. However, at the spinal level CB1Rs primarily regulate Gi/o proteins, and in the absence of Gz proteins, tolerance is primarily achieved by reducing the density of active surface receptors. Indeed, this is seen after repeated systemic treatment with cannabinoids (see Introduction).

## Conclusion

At the supraspinal level, CB1Rs are enriched in neurons, whereas CB2Rs are expressed at very low levels. Brain CB1Rs interact with the HINT1-RGSZ signaling module and produce analgesia by regulating PTX-sensitive Gi/o proteins and PTX-insensitive Gz proteins. A single icv injection of WIN55,212-2, methanandamide, or ACEA brings about a long-lasting tolerance that is not mediated by the persistent loss of functional CB1Rs, but rather, seems to involve the action of CB1R-activated Gαz subunits on certain effector(s). Such effectors appear to be common to CB1Rs and MORs, given their joint regulation of Gz proteins and the fact that Gz deregulation results in analgesic desensitization to cannabinoids and morphine. Together with the findings of other groups, our results suggest that both CB1Rs and MORs co-exist in certain neurons within the brain, where they regulate Gz proteins and similar effectors.

## Methods

### Drugs and production of antibodies

Arachidonyl-2¡-chloroethylamide (ACEA, Tocris #1319), WIN55,212-2 mesylate (Tocris #1038), N-(piperidin-1-yl)-5-(4-iodophenyl)-1-(2,4-dichlorophenyl)-4-methyl-1H-pyrazole-3-carboxamide (AM251, Tocris #1117), and Δ^9^-tetrahydrocannabinol (THC, Pharm, Frankfurt, Germany) were dissolved in 1:1:18 (v/v/v) mixture of ethanol: cremophor EL (Sigma Chemical Co., Madrid): physiologic saline. (*R*)-(+)-Methanandamide in Tocrisolve™ 100 (Tocris #1782). [D-Ala^2^, *N*-Me-Phe^4^, Gly-ol^5^]-enkephalin (DAMGO, Tocris #1171), clonidine hydrochloride (Tories #0690), [D-Ala^2^, D-Leu^5^]-encephalin acetate salt (DADLE, Sigma #E7131), and morphine sulphate (Merck, Darmstadt Germany) were prepared in saline. Pertussis toxin (#516562) and Gö7874 (#365252) were purchased from Calbiochem. The antibodies against CB1R used in this study were produced in New Zealand white rabbits (Sigma Genosys). The antiserum CB1-Nt was raised against amino acid residues 53–66 of the receptor (FRGSPFQEKMTAGD), and the antiserum CB-1EL was raised against residues 177–188 of the murine CB1 receptor (DFHVFHRKDSPN; accession code NP_031752). Anti-CB1R IgGs were purified by affinity chromatography using these synthetic peptides coupled to NHS-activated Sepharose 4 Fast Flow (#17-0906-01, GE Healthcare Biosciences) and labeled with biotin (Sigma #B1022) according to the manufacturer's instructions.

### Reduction of G protein and RGS protein expression

To interfere with the expression of the proteins of interest we used synthetic end-capped phosphorothioate antisense oligodeoxynucleotides (ODNs) which have previously been characterized. These were synthesized by Sigma-Genosys Ltd. (Cambridge, UK). In the following sequences, the nucleotides containing the phosphorothioate linkage are marked with an asterisk: 5'-C*T*CGAATCAGTTCG*C*T-3' (16 nt), corresponding to nucleotides 1044–1059 of the murine RGS9-2 mRNA expressed in the CNS (AF125046) [48,52]; 5'-C*C*GAAGAGTCTCCTC*T*T-3' (17 nt), corresponding to nucleotides 281–297 of the murine RGSZ2 gene (AF191555) [47]; 5'-T*G*TAATCTCACCCTTGCTCTCTGCTGGGCCA*G*T (33 nt), corresponding to nucleotides 1033–1065 of the murine Gαz subunit gene (NM_010311) [49,50]; 5'-G*T*GGTCAGCCCAGAGCCTCCGGATGACGCCC*G*A (33 nt), corresponding to nucleotides 477–502 of the murine Gαi2 subunit gene (NM_008138) [49,50]; 5'-C*C*ATGCGGTTCTCATTGTC*T*G-3' (21 nt), corresponding to nucleotides 725–745 of the Gαq/Gα11 gene sequences (NM_008139/NM_010301) [51]; 5'-T*T*GAGCCTTGGCAAT*C*T-3' (17 nt), corresponding to nucleotides 11–27 of the murine PKCI/HINT1 gene (NM_008248) [32]. These sequences showed no homology to any other relevant cloned proteins (GenBank database). Antisense ODN controls consisted of mismatched sequences in which five bases were switched without altering the remaining sequence.

### Animals, icv injection, and evaluation of antinociception

Male albino CD-1 mice (Charles River, Barcelona, Spain) weighing 22–25 g were housed and used strictly in accordance with the guidelines of the European Community for the Care and Use of Laboratory Animals (Council Directive 86/609/EEC). Animals were kept at 22°C and were on a 12-h light/dark cycle (lights on from 8 a.m. to 8 p.m.). Food and water were provided *ad libitum*. Animals were lightly anaesthetized with ether, and the different substances were injected into the lateral ventricle in a volume of 4 μL as previously described [49]. The response of the animals to nociceptive stimuli was assessed using the warm water (52°C) tail-flick test. Baseline latencies ranged from 1.5 to 2.2 seconds, and they were not significantly affected by the kinase inhibitor Gö7874, its solvent, or the solvent used for the cannabinoid agonists: Gö7874 in DMSO, 1.7 ± 0.1 seconds; DMSO alone, 1.8 ± 0.12 seconds (n = 10); saline, 1.8 ± 0.2 seconds; and ethanol/cremophor EL/physiologic saline (1:1:18), 1.9 ± 0.2 seconds (n = 10). A cut-off time of 10 seconds was used to minimize the risk of tissue damage. Treatment with the selected active and mismatched ODNs did not alter the baseline latencies. Since the mismatched ODNs produced no changes in cannabinoid/opioid activity compared to saline-treated mice, the results obtained with these ODNs are presented as controls. Antinociception is expressed as a percentage of the maximum possible effect (MPE = 100 × [test latency-baseline latency]/[cut-off time-baseline latency]). Groups of 10–15 mice received a dose of cannabinoid agonist and antinociception was assessed at different time intervals thereafter.

ODN solutions were prepared in the appropriate volume of sterile water immediately prior to use. Animals received either vehicle (control), the mismatched sequence ODN, or the antisense oligo. These compounds were injected into the right lateral ventricle. On days 1 and 2, 1 nmol was injected; on days 3 and 4, 2 nmol was injected; and on day 5, 3 nmol was injected. On day 6, the drugs were injected icv and antinociceptive effects were evaluated using the warm water tail-flick test. This schedule of administration did not alter the normal behavior of the mouse [69].

### Production of acute tolerance

Animals were injected icv into the right lateral ventricle with a dose of cannabinoid agonists sufficient to produce 70–80% of the maximum analgesic effect (priming dose). The controls were given vehicle in the same manner. The development of acute tolerance was monitored once the priming dose was observed to have no effect on baseline latencies. Thus, at 24 h an identical dose of agonist (test dose) was administered icv to all the mice – both the treatment and the control groups. Acute tolerance assays were performed when the compound reached its peak effect after 10 min.

### Astroglial cell cultures

Primary mixed glial cultures were prepared from 1-day-old Wistar rat cortex following the procedure described previously [70]. The cultures were maintained for 12 days in DMEM + 10% FCS in a moist 5% CO_2 _atmosphere at 37°C. Enriched astrocytes cultures were obtained after overnight shaking to minimize oligodendrocyte and microglial contamination. For immunoprecipitation studies, astrocytes grown in 75 cm^2 ^flasks were pooled and homogenized in 10 volumes of 25 mM Tris-HCl (pH 7.4), 1 mM EGTA, and 0.32 M sucrose supplemented with a protease inhibitor cocktail (Sigma #P8340, Madrid, Spain). The homogenate was centrifuged at 1000 *g *for 10 min (Sorvall RC5C, rotor SS-34, Newton, CT, USA). The supernatant (S1) was removed and centrifuged at 20,000 *g *for 20 min to obtain the crude membrane pellet and the cytosolic fraction.

For immunocytochemistry, the astrocytes were plated onto poly-D-lysine-coated 10-mm glass coverslips at a density of 20,000 cells/well. After replating, cultures were maintained in DMEM + 10% FCS for 6 h and the serum was reduced to 1% for no longer than 72 h. To label surface CB1 receptors in living astrocytes, cells were incubated with WIN55,212-2 for 1 h at 37°C., after which CB1R antibodies (1:500) labeled with Alexa-488 were added to the cultures. After 30 min the coverslips were rinsed several times with 0.1 M phosphate-buffered saline (PBS) and fixed with 4% paraformaldehyde in PBS for 7 min.

To label internalized CB1 receptors the cells were first incubated with WIN55,212-2 for 1 h at 37°C. Afterwards, the coverslips were rinsed several times with 0.1 M phosphate-buffered saline (PBS) and fixed with 4% paraformaldehyde in PBS for 7 min. This was followed by 45 min incubation with 0.5% NGS, 1% BSA and 0.1% Triton X100. The CB1R antibodies were incubated in this solution for 2 h at room temperature. Cells were observed with a Leica DMIII 6000 CS confocal fluorescence microscope equipped with a TCS SP5 scanning laser. Selectivity of the immunosignal was confirmed by incubating the antibodies with the antigenic peptide.

### Preparation of membranes from neural cells and subcellular fractionation

This procedure has been described elsewhere [25,48]. Briefly, synaptosomal membranes were obtained from groups of 6 to 10 mice that were sacrificed by decapitation at various intervals after receiving icv injection of the compounds. The PAGs were collected and homogenized in 10 volumes of 25 mM Tris-HCl (pH 7.4), and 0.32 M sucrose supplemented with a phosphatase inhibitor mixture (Sigma, P2850), H89 (Sigma, B1427) and a protease inhibitor cocktail (Sigma, P8340). The homogenate was centrifuged at 1000 *g *for 10 min to remove the nuclear fraction. The supernatant (S1) was centrifuged twice at 20,000 *g *for 20 min to obtain the crude synaptosomal pellet (P2). The final pellet was diluted in Tris buffer supplemented with a mixture of protease inhibitors (0.2 mM phenylmethylsulphonyl fluoride, 2 μg/mL leupeptin, and 0.5 μg/mL aprotinin) before aliquoting and freezing at 80°C. The supernatant (S2) was centrifuged at 105,000 *g *for 1 h to obtain the crude microsomal pellet (P3) (Beckman XL-70 ultracentrifuge, rotor Type 70 Ti). The S3 supernatant was concentrated on an Amicon Ultra-4 centrifugal filter device (nominal molecular weight limit [NMWL] of 10,000; #UFC8 01024, Millipore Iberica S.A., Madrid, Spain), and it was then loaded on a 10–40% (w/v) continuous sucrose gradient and centrifuged at 225,000 *g *for 18 h. Ten fractions (4 mL each) were collected, the proteins concentrated, and the CB1Rs immunoprecipitated and analyzed by Western blotting.

### Glycoprotein purification by wheat germ lectin affinity chromatography

Solubilization and wheat germ lectin (WGL) affinity chromatography were carried out at 4°C on neural membranes resuspended in buffer A with 2% Triton X-100 (20 mM Tris-HCl, pH 7.5, 1 mM EGTA, supplemented with protease inhibitor cocktail). The mixture was incubated at 4°C for 16 h with agitation and then centrifuged at 100,000 *g *for 1 h. The clear supernatant obtained was applied at a rate of 1.5 mL/min to a WGL-Sepharose 4B column (GE Healthcare Biosciences, #17-0444) previously equilibrated with 20 bed volumes of buffer A containing 1% Triton X100, 1 mM CaCl_2_, and 1 mM MnCl_2 _(buffer B). The retained glycoproteins were then eluted with 0.25 M N-acetyl-D-glucosamine in buffer B and were collected in siliconized tubes in 1-mL fractions.

### Co-immunoprecipitation of signaling proteins

Samples were sonicated (two cycles of 5 s each) in a volume of 400 μL containing 50 mM Tris-HCl (pH 7.7), 50 mM NaCl, 1% Nonidet P-40, and 50 μL of protease and phosphatase inhibitor mixtures and H89. CB1Rs were immunoprecipitated as described for MORs [25,32,48]. Pilot assays were carried out to optimize the amount of IgGs and sample protein, as well as the period of incubation needed to precipitate the desired protein in a single run. At the end of the procedure, proteins in the soluble fraction were concentrated in centrifugal filter devices (Amicon Microcon YM-10 #42407, Millipore) and solubilized in 2× Laemmli buffer containing mercaptoethanol by heating at 100°C for 3 min. After the samples cooled, proteins were resolved by 10–16% SDS/PAGE.

### Detection of signaling proteins in mouse brain: electrophoresis and immunoblotting

Western blots were probed with affinity-purified IgGs: antibodies directed against peptide sequences in the murine CB1R, i.e., CB1-Nt and CB1-1EL (diluted 1:1000); anti-Gαi1, anti-Gαi2, anti-Gαz (1:2000), anti-Gαo and anti-Gαq/11 (diluted 1:1000) [49,69]; anti-RGS20(Z1) (1:1000) [54], anti-RGS17(Z2) [47], anti-MOR and anti-DOR (1:3000) [45]; anti-PKCI/HINT1 (1:1000) [71]; and anti-PKCγ (1:1000; BD Biosciences). The antibodies were diluted in TBS + 0.05% Tween 20 (TTBS) and incubated with the PVDF membranes for 24 h at 6°C. The primary antibodies were detected using the corresponding secondary antibodies conjugated to horseradish peroxidase (diluted 1:10,000 in TTBS). Antibody binding was visualized with Immobilon Western Chemiluminescent HRP substrate (Millipore #WBKLS0100), and the chemiluminescence was recorded with a ChemiImager IS-5500 (Alpha Innotech, San Leandro, California) equipped with a Peltier-cooled CCD camera that provided a real-time readout of 30 frames per second (-35°C; high signal-to-noise ratio; dynamic range of up to 3.4 optical density units). Densitometry was performed using Quantity One Software (BioRad) and expressed as the mean ± S.E. of the integrated volume (average optical density of the pixels within the object area/mm^2^). The assays were typically performed two to three times on samples obtained from independent groups of mice (n = 12), and the results were always similar.

### [^35^S]GTPγS Binding Assays

Agonist-stimulated [^35^S]GTP*γ*S binding was assayed as described previously [48]. Briefly, synaptosomal membranes from mouse PAG (5 *μ*g of protein) were incubated for 120 min at 30°C in assay buffer containing 50 mM Tris-HCl (pH 7.4), 100 mM NaCl, 10 mM MgCl_2_, 1 mM EDTA, 1 mM dithiothreitol, 10 μM GDP, 0.1% bovine serum albumin, and 0.1 nM [^35^S]GTP*γ*S, together with varying concentrations of WIN55,212-2 (0.1–10 μM). Nonspecific [^35^S]GTP*γ*S binding was assessed by carrying out the above reactions in the presence of 20 μM unlabeled GTP*γ*S. The incubation was terminated by rapid filtration under vacuum through Whatman GF/B filters, followed by three washes with 3 ml of ice-cold 50 mM Tris-HCl (pH 7.2). Bound radioactivity was determined by liquid scintillation spectrophotometry using a Beckman LS-6500 scintillation counter.

### Statistics

All statistical analyses were performed using ANOVA with a Student-Newman-Keuls posthoc test (SigmaStat, SPSS Science Software, Erkrath, Germany), and significance was defined as *P *< 0.05.

## Abbreviations

CB1R: cannabinoid type 1 receptor; MOR: μ-opioid receptor; DAMGO: [D-Ala^2^, *N*-MePhe^4^, Gly-ol^5^] encephalin; PAG: periaqueductal grey matter; RGS: regulator of G-signaling protein.

## Competing interests

The authors declare that, except for income received from our primary employer "Ministerio de Ciencia y Tecnología", no financial support or compensation has been received from any individual or corporate entity over the past three years for research or professional service and there are no personal financial holdings that could be perceived as constituting a potential conflict of interest.

## Authors' contributions

JG conceived the study, participated in its design, and performed the characterization of the CB1R antibodies. MRM and ETM executed the molecular studies, and contributed to the analysis of the data. PSB performed the behavioral studies and assisted with the data analysis and interpretation. JG and PSB wrote and revised the manuscript. All authors have read and approved the final manuscript.
